# Closing the Achievement Gap through Modification of Neurocognitive and Neuroendocrine Function: Results from a Cluster Randomized Controlled Trial of an Innovative Approach to the Education of Children in Kindergarten

**DOI:** 10.1371/journal.pone.0112393

**Published:** 2014-11-12

**Authors:** Clancy Blair, C. Cybele Raver

**Affiliations:** Department of Applied Psychology, Steinhardt School of Culture, Education, and Human Development, New York University, New York, New York, United States of America; The University of Western Ontario, Canada

## Abstract

Effective early education is essential for academic achievement and positive life outcomes, particularly for children in poverty. Advances in neuroscience suggest that a focus on self-regulation in education can enhance children’s engagement in learning and establish beneficial academic trajectories in the early elementary grades. Here, we experimentally evaluate an innovative approach to the education of children in kindergarten that embeds support for self-regulation, particularly executive functions, into literacy, mathematics, and science learning activities. Results from a cluster randomized controlled trial involving 29 schools, 79 classrooms, and 759 children indicated positive effects on executive functions, reasoning ability, the control of attention, and levels of salivary cortisol and alpha amylase. Results also demonstrated improvements in reading, vocabulary, and mathematics at the end of kindergarten that increased into the first grade. A number of effects were specific to high-poverty schools, suggesting that a focus on executive functions and associated aspects of self-regulation in early elementary education holds promise for closing the achievement gap.

## Introduction

The 30th anniversary of the groundbreaking report, A Nation at Risk, serves as a powerful reminder of the persistence and growth of SES-related gaps in achievement [1] and signals the need for a renewed commitment to early learning, particularly for children in poverty. Recent advances in neuroscience suggest that poverty-related gaps in achievement are accompanied by poverty-related differences in brain structure and function [2]–[4] and differences in the regulation of attention, emotion, stress response physiology, and executive functions important for early learning [5], [6]. These findings support the hypothesis that SES-related gaps in academic abilities at school entry are in part attributable to effects of poverty on children’s self-regulation development [7]–[9].

To date, decisions about the most effective ways to foster learning in early childhood have not fully capitalized on advances in the neuroscience of self-regulation: Few interventions or approaches have targeted children’s executive functions and self-regulation, despite evidence of the plasticity and malleability of neural and physiological systems that support self-regulation and early learning [10]–[12].

Research has shown that executive functions, defined as cognitive flexibility, working memory, and inhibitory control are malleable [13], [14] and predict academic achievement in children over and above IQ and socioeconomic status [15], [16]. Although executive functions are defined by a specific set of cognitive skills, they are one aspect of a larger self-regulation system consisting of multiple components arrayed along a continuum from effortful to automatic [17], [18]. As a higher order construct embodying the volitional, active engagement of attention and emotion for the purpose of goal-directed action, executive functions can be understood to regulate activity in lower level neural systems associated with attention, emotion, and physiological responses to stimulation. This top-down influence of executive functions is their hallmark and is frequently emphasized in theory and research on cognitive control [19]–[22]. The relation of executive functions to lower level systems, however, is reciprocal. Changes in emotional and attentional state in response to stimulation are accompanied by physiological changes as indicated by circulating levels of catecholamines, dopamine and norepinephrine, and the steroid hormone cortisol that influence and can at high levels overwhelm neural activity in prefrontal cortex, the seat of executive function abilities [23], [24]. Although executive functions can and do exert ‘top-down’ influence on lower-level systems, these cognitive abilities are dependent on and controlled by the ‘bottom-up’ influence of these lower-level systems. This is particularly the case for young children in whom executive function skills are just developing. Educational approaches that foster the development of the self-regulation system, including the regulation of attention, emotion, and stress response physiology can be expected to enhance executive functions and thereby promote learning and beneficial educational outcomes.

Prospective longitudinal research examining relations among early poverty-related adversity and neuroendocrine and neurocognitive function has shown that the stress response can be altered and executive functions compromised for children in severe poverty or facing other early adversity [8], [9], [25], [26]. Few studies, however, have considered ways that the neurobiology of self-regulation can inform educational practice for all children, particularly those from low-income homes [27], [28]. Across several clinical interventions targeting small samples of children facing high environmental risk, results provide promising evidence of benefits to executive functions, brain activity, and stress response physiology, primarily as indexed by levels of salivary cortisol, the end product of the hypothalamic-pituitary-adrenal [HPA] axis [29]–[33]. No prior studies of which we are aware, however, have explicitly built on the neurobiology of self-regulation to test hypotheses about the best ways to structure educational experiences for children.

Here, we focus on kindergarten, an educational opportunity publically available to all age-eligible children within most school districts and 42 out of 50 states, serving over 4 million children annually [34]. Carefully structured opportunities in kindergarten that assist young children in reflecting on knowledge and purposefully and intentionally engaging with learning materials are likely to promote optimal patterns of neuroendocrine and neurocognitive function important for learning. Such opportunities hold substantial promise for reducing or ameliorating poverty-related gaps in school readiness and early school achievement. Accordingly, we hypothesized that educational practices designed to support the development of self-regulation will lead not only to higher academic achievement but will also be associated with beneficial change in measures of children’s attention, executive functions, and stress response physiology. Further, we hypothesized that these effects would be largest for or perhaps even specific to children in high-poverty schools. Children from poverty backgrounds are less well prepared for school both academically and in terms of self-regulation [35], [36]. Effective education approaches that focus on self-regulation in combination with academic abilities could provide substantial educational benefits for children facing early disadvantage [37].

### Description of the Approach

The aim of this study was to marshal advances in neuroscience research and theory [38], [39] to examine the hypothesis that a focus on executive functions and on the regulation of attention, emotion, and stress response physiology that support executive functions can foster children’s educational progress. To meet this aim, we used an experimental research design to test the impact of an innovative educational approach known as Tools of the Mind. This study is the first to examine the kindergarten version of the program. Findings from prior evaluations of the prekindergarten version of Tools of the Mind, however, are mixed. A randomized controlled trial of the prekindergarten version of the program in a high-poverty sample found that the program was effective in promoting executive function abilities [40] with some limited effects on aspects of children’s academic abilities [41]. Another larger trial of the prekindergarten program with a more diverse sample found no effects on any measures of executive functions or academic outcomes [42].

From a theoretical standpoint, expectations for effects of the Tools of the Mind program on educational outcomes in young children are strong. Building on the fundamental insights of Lev Vygotsky, Alexander Luria, and post-Vygotskian scholars [43]–[45], the program was developed over 18 years of intensive effort in preschool and kindergarten classrooms across the United States, and other countries including Chile and Canada. The pedagogical approach includes the use of specific tactics to support memory and learning, and the organization of “shared cooperative activity” designed to promote social-emotional as well as cognitive development. Specifically, Tools of the Mind has a coherent focus on executive functions as a primary mechanism through which children make academic progress and develop social competencies. In doing so, the program blends teacher-led scaffolding of a comprehensive curriculum of literacy, mathematics, and science activities aligned with the Common Core Standards with child-directed activities and structured sociodramatic play. In Tools of the Mind, teachers learn how to organize and manage instruction so that children build self-regulation skills through purposeful interactions with classmates. In this way, the program is designed to foster attention and emotion regulation skills in ways that support the development of executive functions and lead to advances in social and emotional competence. Teachers also engage in daily dynamic assessment of children’s development in core areas and provide individualized, differentiated scaffolding [meaning that teachers increase or decrease instructional and emotional support depending on how a child independently performs a specific skill.] Children in Tools kindergarten classrooms also meet with the teacher to create and follow weekly individual Learning Plans and set and review weekly individual learning goals. In these conferences, children “talk through” both correct and incorrect answers leading to the ability to reflect on and correct mistakes. Children reflect on their learning and develop a sense that ability is controllable by effort, and mistakes and errors are something to learn from, rather than to be dismissed or avoided. Such activities support intentionality and planning and are specifically designed to prompt the use of metacognitive strategies and reflective thinking.

There are a number of major differences between Tools and traditional kindergarten. Primary among these is the use intentional make-believe play. To make play an effective educational approach, play in Tools of the Mind classrooms is planned in advance, includes children’s enactment of roles with implicit rules, role speech, and the use of symbolic props [e.g. a block is a loaf of bread, a brick, a phone, etc.]. Play in kindergarten is distinguished from play in preschool through dramatization, meaning that it is tied to stories and literature rather than grounded in children’s everyday experiences. Tools kindergartens first use fairy tales to support the development of high-level dramatization, and then quickly move into chapter books, dramatizing the life and times of the book one chapter at a time. In this way, intentional pretend play serves as a vehicle to develop language, text comprehension, vocabulary skills, and creativity in children.

Another major difference between Tools and traditional kindergarten is the intentional use of peer interaction and teacher scaffolding to support reflective, higher-order thinking skills and to assist children in developing social competence and intrinsic motivation to accomplish academic tasks and goals. Maintaining and building children’s intrinsic motivation to learn instead of using external rewards [stickers or stars] is a distinguishing feature of Tools. Social interaction within learning activities and games is designed so that children work primarily in pairs with materials that are self-correcting and prompt children to help one another. These interactions are hypothesized to build a classroom community that is conducive to moderate positive emotion arousal, optimal physiological engagement, and thoughtful self-reflection. They are also understood to help children to develop a sense of self-efficacy and an attitude toward the value of task persistence when confronted with difficulties.

### Summary

The teaching practices and activities of Tools of the Mind are designed to promote academic learning and ability by broadly focusing on multiple aspects of self-regulation including executive functions, social and emotion regulation skills, the control of attention, and the regulation of stress response physiology. As such, the key question tested in this study is whether the set of classroom practices embodied in the Tools of the Mind program will lead to academic gains as well as gains in a key set of indicators that reflect neurobiological as well as academic benefit. As such, this study tests questions regarding the malleability of neuroendocrine and neurocognitive function in educational contexts as well as academic achievement in response to an innovative educational approach. We test these questions in ways that are designed to have clear implications for educational policy as well as basic science research addressing the best ways to reduce or eliminate the school readiness gap associated with poverty in a large and diverse sample.

## Methods

### Ethics Statement

All research procedures and protocols including participant recruitment materials were reviewed and approved by the University Committee on Activities Involving Human Subjects at New York University. Parents of participating children provided written consent and all participating children provided verbal assent.

### Participants and Procedures

School districts were contacted by research and program personnel and recruited into the study. Twelve districts with a total of 29 schools agreed to participate. Schools signed a memorandum of understanding in which they agreed to accept random assignment to the treatment or control group, to remain in the assigned condition for two years, to facilitate data collection, and if randomly assigned to the treatment group to facilitate the training and coaching. Schools were blocked according to district, percent of students eligible for free or reduced-price lunch, school size, state test scores, and number of kindergarten classrooms, and randomly assigned to the treatment [N = 16] or control [N = 13] group. Block randomization on multiple variables led to small and acceptable differences in the number of schools randomized to the treatment vs. control groups. Most schools included two participating kindergarten classrooms. We made an exception for 2 schools in the participating districts in which all of the district’s kindergarten classrooms were housed in one facility. For these two schools, random assignment was at the classroom level and the school was treated as two schools for analysis purposes. The sample included a total of 79 classrooms, N = 42 treatment and N = 37 control.

Participating schools ranged from 5% to 92% of students eligible for free or reduced-price lunch. Approximately 15% of the schools in the sample are considered high-poverty schools as defined by the National Center for Education Statistics [46] as schools with greater than 75% of students eligible for free or reduced-price lunch. Approximately 50% of the schools in the sample are considered low-poverty schools, meaning that less than 25% of students are eligible. The remaining schools ranged from 27% to 68% of students eligible for free or reduced-price lunch.

We attempted to recruit six children per classroom in each of the two years of the study. Recruitment was conducted through flyers sent to children’s homes and through parent night activities. Parents provided written consent for children to participate and children provided verbal assent. In instances in which more than 6 children in a classroom expressed interest in participation, we enrolled the first 6 children for whom consent forms were returned by parents and assigned all other children with returned consents to a waiting list. Recruitment efforts yielded a sample of N = 396 in Year 1 [229 treatment, 167 control] and N = 363 in Year 2 [214 treatment, 149 control] for a total analysis sample of N = 759. Children in treatment classrooms were no more likely to participate than were children in control classrooms. Approximately 5% of participants [N = 34] were lost to follow-up from fall to spring and replaced with participants on waiting lists in classrooms in which more than 6 participants expressed interest in participating in the study or in some classrooms with newly recruited participants. Additionally, 2 new participants were recruited in the spring in classrooms in which fewer than 6 children had been recruited in the fall. All children entering the study in the spring had been in the classroom since the fall. Total sample size was N = 723 in the fall and N = 725 in the spring. Fall to spring attrition was unrelated to treatment or control group status.

Children were assessed in the fall and spring by trained data collectors at the school in which the child was enrolled. All measures were administered in a standard order. Assessment sessions on average took less than one hour to complete. In both fall and spring, children participated in two test batteries, one of which included measures of executive function, attention, and speed of processing, and the other measures of academic ability and general reasoning and during which 3 saliva samples were collected to assess physiological reactivity to the assessment session. The timing of saliva collection was designed to assess cortisol reactivity to the testing session. Given that the expression of cortisol in saliva generally lags any change in HPA activity by approximately 20 minutes, we collected an initial sample within 5 minutes of meeting the child for the testing session and then collected second [post1] and third [post2] samples at 20-minute intervals. The first sample provides an indication of the child’s physiological state in the classroom prior to the testing session rather than a true baseline or resting state and the post1 and post2 samples provide indications of the child’s response to meeting the research assistant and participating in the battery.

On average children were seen at the end of October in the fall and at the end of March in the spring. The second assessment session occurred approximately two weeks after the first in both fall and spring. The average number of days between fall and spring assessments was M = 156 [SD = 27]. The treatment and control groups did not differ in the number of days between the fall and spring assessments.

All participating children were followed up in the fall of the first grade year. Children were seen by trained data collectors in a single session at the school in which they were enrolled and administered measures of academic ability.

### Treatment Classrooms

Teachers and teaching assistants in the *Tools of the Mind* classrooms were trained in a 2-year professional development cycle. In Year 1 teachers had 4 workshops spread across the year with a total of 5 days of training [Workshop 1 is 2 days]. Year 2 had 3 training workshops spread across the year with 3 days of training. Each school had a Tools coach that worked with the Tools trainer to provide in-classroom coaching once every other week during Year 1 and then once a month in Year 2. Coaches varied in their experience with Tools. Most were new to Tools and trained with the teachers during Year 1. There were self-reflection forms for the teacher to complete to assist the teacher in thinking about the implementation of different activities and to help the teacher reach better fidelity. Four times a year the Tools trainer would visit classrooms with the Coach to give feedback to the coach. Tools teachers may have attended district-wide professional development, such as health-related training, but they were not trained on any other curriculum during the course of the study.

### Control Classrooms

Schools randomly assigned to the control condition continued with ‘business as usual’ practice. For example, teachers in control classrooms continued with district professional development as ordinarily scheduled through the school. Classrooms in the control schools used a combination of commercial literacy and mathematics curricula and followed state standards for the development of science and social studies curricula, a typical scenario for kindergarten classrooms. Tools and control classroom curricula meet Massachusetts State Standards and are aligned with the Common Core Standards. No classrooms in the control condition contained activities resembling those in the *Tools of the Mind* classrooms. For example, in the control classrooms children’s make-believe play was not intentionally nurtured or scaffolded. In traditional, academically-oriented kindergartens, play is commonly relegated to a 10–15 minute free choice time at the beginning and ending of the school day or during recess, and many classrooms no longer have materials to support children’s engagement in make-believe play. Most importantly, none of the literacy curricula used in control classrooms promote the use of literature-based make-believe play as a vehicle for teaching new vocabulary and text comprehension. While some of these curricula may suggest using elements of play in literacy activities it is never done in a systematic fashion across different titles and text genres.

### Measures

The measures of academic ability included the Applied Problems and Letter-Word subtests from the Woodcock-Johnson III Tests of Achievement [47]. The WJ III is a co-normed set of tests for measuring general scholastic aptitude, oral language, and academic achievement. The validity and reliability of the WJ III Tests of Achievement is well established [48]. To measure fall to spring change and follow-up in the first grade, we used W scores on each of the subtests as these scores are most appropriate for examining individual level change over time [49]. To assess vocabulary ability in the spring and in the first grade follow-up we used the Expressive One Word Picture Vocabulary Test [50] raw score. The EOWPVT is a norm-referenced measure of vocabulary that assesses the child’s ability to use words and requires the child to access and retrieve words from memory. The internal consistency reliability of the measure is .98. For the fall [pretest] measure of vocabulary, we used the raw score of the Reading Vocabulary subtest of the Woodcock-Johnson III Tests of Achievement.

The measure of general reasoning was the Raven Colored Progressive Matrices test [51]. The Raven test is a measure of fluid intelligence in which children are presented with a series of plates containing patterns from which a single uniformly shaped piece is missing. At the bottom of each plate, six versions of the missing piece are presented, only one of which correctly fits the pattern. Children were presented with a demonstration trial and then instructed on successive trials to point to the piece that best completes the pattern. Children were presented with one set of 12 plates of increasing difficulty. Total number correct was used for analysis.

Measures of executive function abilities were comprehensive and included assessments of working memory, cognitive flexibility, and inhibitory control. Three measures of executive function were administered on a laptop computer, including the Hearts and Flowers task [52], the Flanker with Reverse Flanker task [40], and the NIH Toolbox version of the Dimensional Change Card Sort task [53]. Children were instructed to respond by pressing one of two designated keys on opposite sides of the keyboard to which small Velcro strips were affixed to cue children to the location of the keys.

On the Hearts and Flowers task, children are instructed to respond by pressing the designated key on the same side as the stimulus when the stimulus is a heart and on the opposite side when the stimulus is a flower. Children are presented with instructional and practice trials, which can be repeated up to three times if necessary, followed by 12 hearts only trials, 12 flowers only trials, and 33 trials on which hearts and flowers are intermixed.

On the Flanker with Reverse Flanker task children are shown a row of five fish that are either blue or pink. When the fish are blue, [the standard flanker block], children are instructed to only pay attention to the middle fish and to press the designated key on the side of the keyboard to which that fish is facing. When the fish are pink [the reverse flanker block], children are instructed to only pay attention to the outer four fish, the flanker fish, and press the designated key on the side of the keyboard to which those fish are facing. On congruent trials, the central fish and the flanking fish are facing the same direction, and on incongruent trials, the central fish and flanking fish are facing in opposite directions. Children are presented with instructional trials and practice trials, which can be repeated up to 3 times and then presented with 50 trials on which congruent and incongruent responses to pink or blue fish are intermixed. In the fall [pretest], children were administered the standard version of the flanker task [54]. In the standard version of the task, the fish are all similarly colored and children respond only to the direction that the middle fish is facing.

On the NIH Toolbox version of the DCCS, children are presented with instructional and practice trials and then complete the standard version of the task, sorting 6 images by color and then by shape. Following this children are presented with 50 mixed trials in which the instruction to sort by color or by shape is presented auditorily.

All computer administered executive function tasks were scored as percent correct responding from which total percent correct and percent correct on incongruent relative to percent correct on congruent trial scores were derived. Reaction time/response latency data were also collected. Latencies less than 200 ms or greater than 3 standard deviations above the mean were excluded. Mean response latencies were calculated for each trial type if at least 60% of trials were valid.

The executive function of working memory was assessed in the spring [posttest] using the Forward/Backward Digit Span task [55]. This task was modeled on Weschsler [56]. Children are presented with digit strings of increasing length in two-trial sets beginning with two-item sets and progressing through three-, four-, and five-item sets. Children are first instructed to repeat the digits spoken by the experimenter in the order that the experimenter speaks them. Forward digit span continues until the child fails both item sets at a given trial level. The experimenter then asks the child to continue the task but to repeat the digits spoken by the experimenter in reverse order. As with forward span, digit strings of increasing length are presented in two-trial sets beginning with two two-item sets and progressing through two three, four, and five item sets. Testing continues until the child fails both item sets at a given trial level. For both the forward and backward version of the task children are given instructional and practice trials. The task was scored as total number correct items sets. To control for baseline ability in the fall [pretest], we used a z-score composite of percent correct responding on the executive function tasks.

We assessed the ability to control attention in the face of emotional distraction using a Dot-Probe task, a widely used measure of attention bias to emotional stimuli [57]. Children are instructed to respond using the designated key on the keyboard corresponding to the side of the computer screen on which a stimulus [a dot] appears. A fixation cross is presented in the center of the screen for 750 ms followed by two pictures presented simultaneously for 750 ms on the left- and right-hand sides of the computer screen. Pictures included images drawn from the International Affective Picture database. For each pair of pictures, a neutral picture [ex. chair, lamp, cup] was paired with another neutral picture or with an emotionally arousing picture [ex. snakes, wolves, car crash]. Following the picture presentation, a dot either appears in the same location as the emotionally arousing image [congruent trials], in the location opposite the threatening image [incongruent trials], or on either side of the neutral/neutral display [neutral control]. The dot remains on the screen for 5000 ms or until the participant responds. Following instructional and practice trials, forty test trials were presented in a semi-random order that was the same across all participants. Trials with latencies less than 100 ms or greater than 3 standard deviations were excluded from analyses. Mean response latencies were calculated for each trial type if at least 60% of trials were valid. Negative response bias was calculated by subtracting the mean latency to respond to congruent trials from the mean latency to respond to incongruent trials. If participants attend to the emotion image, latencies will be faster for congruent displays and longer for incongruent displays and bias scores will be large and positive. Conversely, if participants are less distracted by the emotion image, the difference between the latencies will be shorter and the difference will be smaller.

Indicators of neuroendocrine function, cortisol and alpha amylase, were obtained through unstimulated whole saliva collected using hydrocellulose absorbent material and expressing sample by centrifugation. Samples were frozen at −20 C prior to being shipped to the laboratory packed in dry ice, and subsequently frozen at −80 C. All samples were assayed for salivary cortisol using a highly sensitive enzyme immunoassay [58]. The test used 25 µl of saliva, had a range of sensitivity from 0.007 to 3.0 µg/dl, and average intra- and inter- assay coefficients of variation less than 10% and 15%, respectively. All samples were assayed in duplicate and the average of the duplicates was used in all analyses. Natural log transformations were applied to the cortisol values to correct for positive skew.

Samples were also assayed for alpha amylase using a commercially available kinetic reaction assay [58]. The assay employs a chromagenic substrate, 2-chloro-pnitrophenol, linked to maltotriose. The enzymatic action of sAA on this substrate yields 2-chloro-p-nitrophenol, which can be spectrophotometrically measured at 405 nm using a standard laboratory plate reader. The amount of alpha-amylase activity present in the sample is directly proportional to the increase [over a 2-min period] in absorbance at 405 nm. Results are computed in U/ml of alpha-amylase using the formula: [absorbance difference per minute X total assay volume [328 ml] X dilution factor [200]]/[millimolar absorptivity of 2-chloro-p-nitrophenol [12.9] X sample volume [.008 ml] X light path [.97]]. Square root transformations were used to reduce positive skew.

Finally, we included measures of speed of processing to assess potential program effects on this basic aspect of attention and neural efficiency. Measures of speed included a simple reaction time task and a rapid automatized naming task. In the simple reaction time task [35], children were instructed to press the laptop space bar each time a blue dot [7 cm diameter] appeared at random locations across the bottom of the screen. Responses were recorded in milliseconds. The inter-stimulus interval varied between 500 ms and 2500 ms. Children completed a total of 30 trials. Lower scores represent faster processing speed. Trials with latencies less than 200 ms or greater than 3 standard deviations above the mean were excluded from analyses. Mean response latencies were calculated for each trial type if at least 60% of trials were valid. In the rapid automatized naming task [59], children were presented with a sheet of 8.5×11 in paper across which are four rows containing nine color blocks each composed of blue, black, green, red, yellow, and brown squares in pseudo-random order. The child was instructed to name each color in turn quickly but accurately, starting with colors on the top row moving from left to right and row to row until all of the colors have been named. Following demonstration and practice, the child completes the sheet and is presented with a second sheet and instructed to continue naming the colors. The experimenter records the length of time it takes the child to complete the task. Timing is discontinued if the child makes 4 or more errors and the child is considered to have failed the task.

### Data Analysis

Means, standard deviations, and numbers of participants with data for each variable in the treatment and control groups for each assessment in the fall and spring of kindergarten are presented in Tables 1 and 2. Follow-up data in first grade are also presented for the academic outcome measures in Table 2. As noted above, fall to spring attrition was approximately 5 percent of cases in the sample overall and 34 participants were replaced and two new participants were added to the sample in the spring. Fall to spring attrition did not differ between the treatment and control groups. A number of variables were also missing some data in the fall or spring for various reasons. For the academic measures, Raven matrices, forward/backward span, and simple reaction time measures, fewer than 5% of children were missing data, generally as a result of fatigue or absence from school and the data are considered to be missing at random. For the computer-administered executive function measures [DCCS, Fish Flanker, Hearts and Flowers] and the Dot-Probe task, missing data were primarily due to children having fewer than 60% valid trials and/or reaction times out of the usable range [e.g., <200 msec.] Missing data for cortisol and sAA were due to insufficient volume of saliva collection or child refusal. Missing cortisol and sAA were also due to refusal of the school principal at one of the participating schools to allow saliva collection from children. Missing data for all variables were unrelated to treatment vs. control status. Analyses were run using full information maximum likelihood estimation to adjust for potential bias in the estimates resulting from missing data.

**Table 1:** Unadjusted descriptive statistics and sample size for measures of self-regulation and speed of processing in fall and spring of kindergarten.

	Tools of the Mind				Control			
	Fall		Spring		Fall		Spring	
	N	Mean [SD]	N	Mean [SD]	N	Mean [SD]	N	Mean [SD]
Dimensional Change Accuracy	351	.65 [.13]	327	.73 [.12]	240	.66 [.13]	235	.73 [.14]
Hearts & Flowers Accuracy	390	.70 [.17]	403	.76 [.17]	273	.69 [.19]	284	.75 [.18]
Flanker Accuracy^a^	346	.74 [.20]	––	––	227	.75 [.21]	––	––
Reverse Flanker Accuracy^b^	––	––	342	.56 [.17]	––	––	234	.55 [.17]
EFs Accuracy z-score Composite	383	−.014 [77]	399	−.003 [.76]	263	−.004 [.82]	281	−.03 [.82]
Dimensional Change RT msec	339	1083.2 [154]	322	1067.4 [143]	234	1089.6 [145]	233	1077.6 [139]
Hearts & Flowers RT Mixed msec	361	1185.1 [222]	389	1084.3 [209]	247	1181.6 [218]	267	1111.1[207]
Flanker RT msec^a^	324	1208.9 [209]	––	––	213	1202.8 [202]	––	––
Reverse Flanker RT msec^b^	––	––	329	1420.7 [247]	––	––	224	1438.8 [231]
EFs RT z-score Composite	407	.03 [.85]	413	−.03 [.86	281	−.02 [.82]	288	.05 [.83]
Backward Span Percent Correct^b^	––	––	416	.25 [.13]	––	––	282	.23 [.14]
Raven Matrices Sum Score	418	8.14 [1.9]	413	8.78 [1.5]	289	7.93 [1.9]	293	8.67 [1.7]
Dot-Probe Negative Bias	305	−14.14 [140]	313	−4.70 [124]	206	−18.47 [159]	229	8.25 [124]
Simple Speed RT msec	417	639.1 [212]	420	529.7 [116]	289	638.4 [199]	295	536.4 [124]
Rapid Color Naming Total Time	348	49.83 [17.7]	373	46.35 [14.4]	241	51.50 [16.4]	258	48.39 [17.2]
Sample 1 log Cortisol ug/dl	348	−2.13 [.69]	364	−2.03 [.60]	254	−2.07 [.65]	279	−2.05 [.60]
Sample 2 log Cortisol ug/dl	349	−2.23 [.68]	366	−2.10 [.57]	249	−2.13 [.67]	279	−2.12 [.61]
Sample 3 log Cortisol ug/dl	335	−2.33 [.67]	353	−2.19 [.55]	235	−2.25 [.67]	272	−2.22 [.62]
Sample 1 sq Alpha Amylase	357	8.02 [3.2]	362	7.99 [3.2]	253	7.80 [3.1]	278	8.25 [3.2]
Sample 2 sq Alpha Amylase	352	8.49 [3.2]	365	8.47 [3.3]	251	8.35 [3.2]	278	8.91 [3.4]
Sample 3 sq Alpha Amylase	342	8.43 [3.1]	355	8.44 [3.3]	233	8.26 [3.1]	268	8.83 [3.2]

a fall [pretest] only,

b spring [posttest] only.

**Table 2:** Unadjusted descriptive statistics and sample size for measures of academic outcomes in fall and spring of kindergarten.

	Tools of the Mind						Control					
	Fall K		Spring K		Fall First		Fall K		Spring K		Fall First	
	N	Mean[SD]	N	Mean[SD]	N	Mean[SD]	N	Mean[SD]	N	Mean[SD]	N	Mean[SD]
Applied Problems W Score	408	434.08	410	446.08	393	458.40	282	432.58	289	443.49	268	456.44
		[17.1]		[16.6]		[19.1]		[17.9]		[18.2]		[17.6]
Letter-Word W Score	416	370.16	413	404.66	396	434.04	288	370.44	290	403.22	268	430.81
		[31.1]		[32.1]		[33.3]		[31.2]		[29.9]		[30.2]
Picture Vocabulary Sum^a^	417	12.32	–-	–-	–-	–-	290	12.01	––	––	–-	–-
		[2.1]						[2.1]				
EOWPVT Raw Score^b^	––	––	417	71.73	397	76.93	––	––	297	69.09	270	72.53
				[16.9]		[20.9]				[17.1]		[22.1]

a fall [pretest] only,

b spring [posttest] only.

We analyzed the data using multilevel models with children nested within schools, the unit of random assignment. We bootstrapped clustered standard errors [B = 1000] to provide asymptotic refinement and more consistent estimates of standard errors [se] given that the data are hierarchically nested in a small number of clusters [N = 29] and variances may be heteroskedastic [60], [61]. For all analyses, treatment versus control group status was included as a binary indicator along with the pretreatment [fall] measure of the outcome variable and child-level [age, sex, parent education, pre-treatment cognitive ability] and school-level [percent free or reduced-price lunch, size of kindergarten enrollment] covariates. By including the pretreatment measure of each outcome as a predictor, the test of the coefficient [b] for the treatment versus control group comparison indicates whether Tools of the Mind was associated with greater change from the pretreatment measure in the fall. To make this comparison of fall to spring change explicit, we also examined a change score as the outcome variable, subtracting the fall measure of the outcome from the spring measure and using this as the dependent variable. Given the redundancy of results for these analyses with those we report when adjusting for the pretest measure, we do not report them but make them available from the first author upon request. For all analyses, we first tested the main effect of treatment [Tools of the Mind vs. Control] on the outcome, and then tested the interaction of treatment with the school-level variable, percent of children eligible for free or reduced-price lunch, to determine whether effects were larger in high-poverty schools.

In the two-level model predicting student outcomes, child posttest score on a given assessment [denoted by 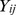] was predicted at level 1 from a vector of child-level predictors including the child’s pre-treatment score on the outcome assessment and child-level covariates.
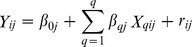



Test of the effect of the treatment on child posttest score was included in the second level of the model as a binary indicator 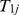 [treatment = 1, control = 0] to account for the nesting of participants within schools, the unit of random assignment. School level covariates were also included in the model as a vector of predictors 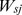 at level 2.
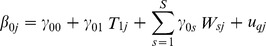



For outcome assessments with three time points, namely, measures of cortisol and alpha amylase, and mathematics and reading ability with first grade data included, we added a within-person level to the model, in addition to child and school levels. In these models we examined treatment effects on the linear slope as well as the intercept. For the prediction of math and reading outcomes, the intercept was set at the last time point, first grade.

Effect sizes were calculated as the covariate adjusted model parameter estimate for the binary treatment versus control comparison divided by the pooled standard deviation for the outcome. This provides an estimate of the covariate adjusted mean difference between groups in standard deviation units. Effect sizes were calculated for all treatment effects observed in two-level models, whether main effects or interactions with school poverty. Effect sizes for the interaction effects were calculated with school percent free or reduced-price lunch equal to 1. Effect sizes were not calculated for treatment effects in three-level models given uncertainty as to how to best to make this calculation.

## Results

We ran analyses on a number of dependent variables to address a theoretically coherent and interrelated set of research questions. We report results of all analyses below, first reporting the test of the main effect of treatment and then the test of the interaction of treatment with the school-level poverty indicator, percent of students eligible for free or reduced-price lunch. As a preliminary step in the analysis and check on randomization, we examined treatment and control group differences on all variables assessed in the fall of kindergarten to determine if differences between the groups were present at baseline. No differences were observed.

**Figure 1:**
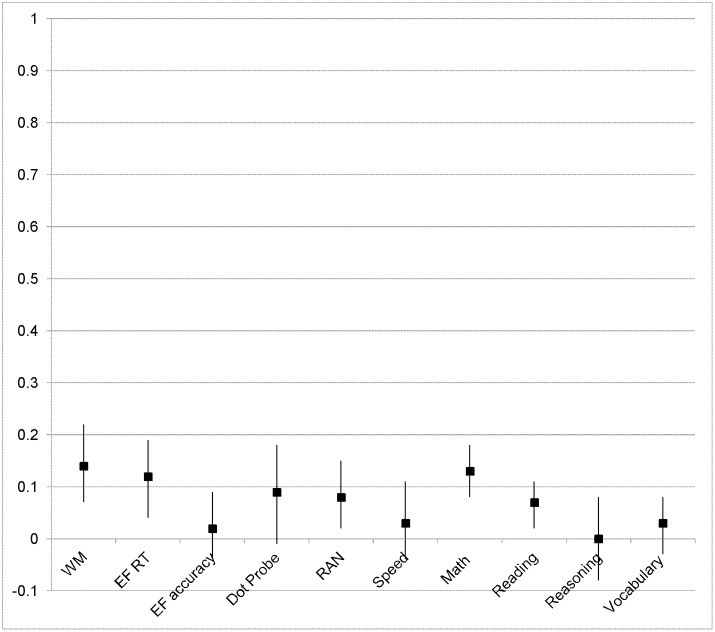
Effect size estimates for main effects of Tools of the Mind versus Control group comparisons at the end of kindergarten. Error bars represent effect sizes at ± 1SE.

A primary question of interest to both education and neuroscience communities is whether the Tools of the Mind program led to significant improvements relative to the control group in children’s neurocognitive and neuroendocrine functioning. Our analysis suggests that the answer is largely yes: Children in classrooms implementing Tools of the Mind did significantly better than children in the control classrooms on a measure of working memory, the backward digit span task, b = 0.02, se = .01, p<.05, ES = .14. The effect size [ES] estimate and 95% confidence interval for the estimate for this comparison and for all outcomes included in the analysis are presented in Figure 1. This effect was observed in the sample as a whole and was not larger in high-poverty schools.

**Figure 2:**
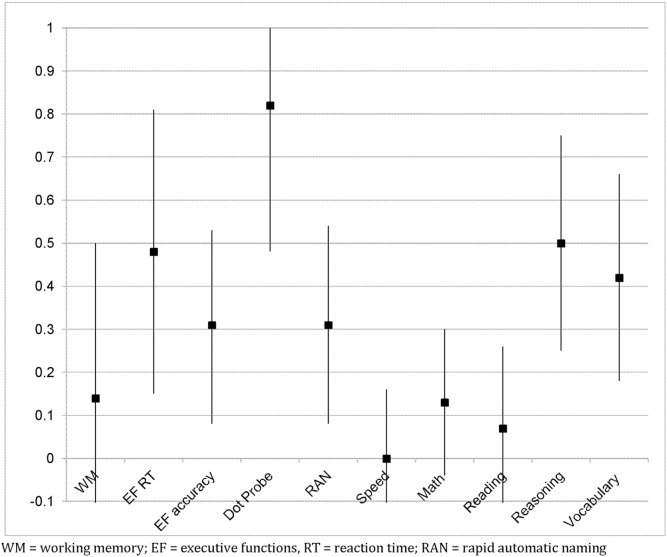
Effect size estimates for Tools of the Mind versus Control group comparisons in high poverty schools at the end of kindergarten. Error bars represent effect sizes at ± 1SE.

Children in classrooms implementing Tools of the Mind also had significantly faster mean reaction time than did children in control classrooms on a composite of inhibitory control and rule switching computer administered executive function tasks including the DCCS mixed trials, the Hearts & Flowers mixed trials, and the Flanker with Reverse Flanker total score, b = −20.62, se = 12.63, p<.05, ES = .12. This effect indicates that children receiving the Tools of the Mind program were better able to cope with cognitive interference and subsequent slowing of responding on these tasks. The analysis of this composite also indicated that the effect of treatment on reaction time tended to be larger in high-poverty schools, b = −75.7, se = 55.9, p = .10, ES = .48. Figure 2 presents the ES estimate and 95% confidence interval for this comparison and for all outcome variables in high-poverty schools. We did not observe an overall effect of Tools of the Mind on accuracy [percent correct responding] on this executive function composite. The effect of the program on accuracy on the executive function composite, however, was large in high-poverty schools although not statistically significant using the conventional threshold, b = .039, se = .029, p<.10, ES = .31.

A further effect of the curriculum on the control of attention in the face of interference was also seen on a task in which emotionally arousing stimuli are interspersed among neutral images and are either congruent or incongruent with a cued response location. On this task, the Dot-Probe, we did not observe an overall effect of Tools of the Mind but found that the effect of the program on this outcome was specific to high-poverty schools. Children receiving Tools of the Mind in high-poverty schools exhibited reduced interference from the emotional image on response latency on incongruent relative to congruent trials, referred to as negative bias, b = −102.2, se = 47.01, p<.05, ES = .82 [Figure 1b]. The effect of the curriculum on the Dot-Probe task was not seen, either in the sample overall or in high-poverty schools for attention as assessed by a simple reaction time task. The test of the effect of Tools of the Mind on the rapid automatized naming task, a measure of speeded responding, however, indicated that children in Tools of the Mind classrooms were more efficient at processing information, b = 1.44, se = .97, p<.05, ES = .08. This effect of the treatment was larger, although not conventionally statistically significant, in high-poverty schools, b = 4.36, se = 4.04, p<.20, ES = .28.

Of further interest, analyses revealed significant impacts of Tools of the Mind on measures of children’s stress response physiology that were specific to high-poverty schools. No main effect of treatment was observed. Levels of cortisol and alpha amylase were assessed through saliva samples collected at three time points over one data collection session [baseline, 20 mins post, and 40 mins post]. Controlling for the time of day of data collection, a three level model with observations nested within participants nested within schools indicated that salivary cortisol levels declined significantly in both groups, b = −.19, se = .03, p<.001, across the testing session but that overall levels were higher, b = .25, se = .07, p<.05, among children in the classrooms implementing Tools of the Mind in high-poverty schools. Similarly, salivary amylase levels increased significantly over the testing session in both groups, b = .48, se = .14, p<.001, but were significantly lower, b = −1.62, se = .38, p<.05, among children in the classrooms implementing Tools of the Mind in high-poverty schools.

Finally, as hypothesized, the analysis demonstrated that Tools of the Mind substantially benefited children’s academic progress relative to the progress of children in the control schools [Figures 1 and 2]. Children in classrooms implementing Tools of the Mind showed significantly higher academic ability than did children in control classrooms on a measure of mathematics, b = 2.10, se = .94, p<.05, ES = .13. This effect of Tools of the Mind on mathematics in the sample overall was not significantly larger in high-poverty schools. In contrast, a large effect of treatment on vocabulary was specific to children in high-poverty schools, b = 8.34, se = 4.30, p<.05, ES = .43. The specific effect of the curriculum in high-poverty schools was also seen on a measure of general fluid intelligence or reasoning, the Raven colored matrices test, b = −0.75, se = .42, p<.05, ES = .46.

**Figure 3:**
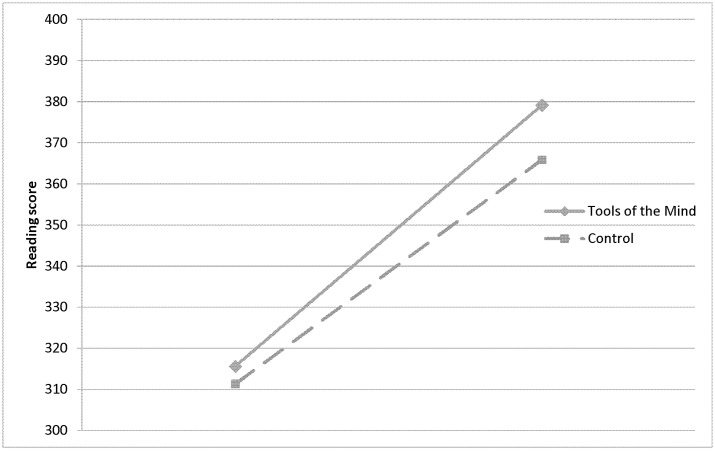
Effect of the Tools of the Mind curriculum on growth in reading (linear slope) from the beginning of kindergarten through the fall of first grade, b = 2.24, se = .83, p = .001.

Follow-up data in the first grade on children participating in the study [see Table 2] indicated that an initially non-significant main effect of the Tools of the Mind program on reading ability, b = 2.00, se = 1.42, p<.10, ES = .07, increased and became significant, b = 3.32, se = 1.45, p<.05, ES = .14. The sustained effect of the program was also observed for vocabulary and extended to all children receiving Tools of the Mind, not only children in high-poverty schools, b = 2.16, se = 1.14, p<.05, ES = .10. Some sustained effect of the program was also observed for mathematics to some extent, b = 1.36, se = 1.01, p<.10, ES = .07, however, the effect of Tools of the Mind on achievement in mathematics was somewhat reduced from that seen at the end of kindergarten. Notably, the effects of the program on progress in reading, vocabulary, and mathematics were detected when controlling for both the pre [fall] and post [spring] measures of the outcome in kindergarten, indicating an effect of the curriculum over and above that seen at the end of kindergarten. These findings indicate that the significant improvement in learning in children in the Tools of the Mind classrooms relative to the control classrooms continued into the first grade. This effect of the curriculum on growth in child academic ability is readily illustrated for reading, for which we have the same measure at all three time points. A three level model with measurement occasion nested within participants and participants nested within schools indicated a positive treatment effect on the slope for reading ability, b = 2.24, se = .83, p<.01 [Figure 3], indicating faster growth in reading ability in the treatment group relative to the control group.

## Discussion

The results of this field-based experiment demonstrate that the Tools of the Mind program, which focuses broadly on the development of self-regulation as key to early learning, led to improvements in neuroendocrine and neurocognitive function as well as significant improvements in academic abilities. These findings indicating positive effects of Tools of the Mind on children’s executive functions and attention as well as on indicators of stress response physiology help to empirically confirm the responsiveness to experience of these aspects of children’s development as well as substantiate the relevance of an educational approach that builds on the neurobiology of self-regulation. No prior study of which we are aware has shown widespread effects of a focus on self-regulation on multiple interrelated aspects of children’s early development in an educational setting using an experimental design. A small number of experimental studies implementing programmatic activities to support self-regulation development have shown effects on stress response physiology as indicated by cortisol [31] and on the control of attention both behaviorally and in terms of brain activity [32]. Our study, however, is the first to comprehensively assess and document treatment-related differences in neuropsychological and psychophysiological measures as well as academic outcomes in a typically occurring educational context.

The fact that these broad experimental effects were observed in response to an innovative program focused on self-regulation development suggests that the approach embodied in Tools of the Mind is effective at engaging children and assisting children in making academic progress. As noted above, prior findings of the prekindergarten version of the program have been mixed. It may be that kindergarten represents an optimal time during which the activities contained in Tools of the Mind can support and foster self-regulation skills and abilities in children. Executive functions and the control of attention enable children to focus on and process information more efficiently while higher levels of cortisol and lower levels of amylase provide evidence of physiological support for engagement. Although stress physiology is complex and lower levels of cortisol and higher levels of alpha amylase are generally desirable at rest, elevated cortisol under conditions of moderate stress and engagement is associated with a higher level of cognitive ability [62], [63]. Moderate cortisol elevations, taking into account diurnal variation, promote memory and learning and facilitate energy utilization and storage [64], [65]. Although sustained diurnal elevations in cortisol are injurious to the organism and indicative of chronic stress, our findings are not consistent with an interpretation in which the relative elevations we observed in children are indicative of increased stress in Tools of the Mind classrooms. Notably, levels of salivary cortisol significantly declined, and levels of sAA significantly increased during the approximately 50 minute assessment session in both groups. On average, children in the sample did not exhibit any indication of the sustained elevated levels of salivary cortisol that are reported in research on children in preschool settings [66]–[68]. Research on stress response physiology in children in elementary education settings is rare and only one other study to our knowledge, with a middle income sample of preschoolers, has examined change in salivary cortisol and sAA using a protocol similar to ours [69]. Consistent with our findings, in that study, lower cortisol across the assessment session was associated with behavior regulation difficulties as rated by teachers and higher levels of sAA were associated with greater difficulty delaying gratification.

Perhaps most importantly, a number of the effects we observed, including effects for salivary cortisol and alpha amylase, are specific to high-poverty schools. Recent research has highlighted the neurobiological and cognitive costs associated with exposure to poverty-related adversity. Our findings highlight that children in high-poverty schools demonstrate significant neurobiological and academic benefit when provided with an educational approach that provides scaffolded support for self-regulation. The indication that such an educational approach can work to overcome deficits in school readiness associated with poverty has substantial implications for school reform and anti-poverty efforts. As well, the indication that academic gains associated with the curriculum are sustained and increase over time into the first grade for all children contributes to the profound expectation that effective early education can work to reduce the achievement gap and thereby help to reduce growing social and economic inequality in the United States while also providing benefits to all children’s learning.

Addressing inequities in educational opportunity associated with poverty is a national priority. Educational disadvantages associated with poverty are present prior to kindergarten [70,]. By improving children’s skills at the outset of their educational careers, our findings suggest that effective child-directed educational approaches such as Tools of the Mind can perhaps go a long way toward fulfilling the promise of free and universal public education by equalizing opportunity for children to succeed despite initial disadvantage. By working to level the playing field for children at school entry, effective kindergarten education can be expected to lead to reduced social and economic burden associated with poverty. Long term follow-ups of model early intervention programs of the 1970s and 1980s indicate that these high quality educational programs resulted in greater academic achievement, school completion, and employment, and reduced grade retention, special education, arrest, and incarceration among program recipients [71]. These findings are complemented by longitudinal findings from a birth cohort of children in Dunedin, New Zealand indicating that self-regulation in childhood, measured between ages 3 and 11 years is a powerful predictor of well being in adulthood [72]. In sum, programs that can improve self-regulation in children can be expected to have long-term benefits.

Although the case is clear for initiating anti-poverty programs as early as possible, far in advance of kindergarten entry, kindergarten represents an excellent window of opportunity for implementing the child-focused approach embodied by Tools of the Mind. Educational practice increasingly emphasizes academic content in kindergarten, and children of kindergarten age are for the most part ready to engage with that content. There is, however, surprisingly little research and policy emphasis on kindergarten effectiveness. This is despite the indication that a high quality kindergarten experience may uniquely influence later educational and life outcomes. Evaluation of the kindergarten class size experiment in Tennessee known as Project Star indicated that students randomly assigned to kindergarten classrooms with fewer students had higher academic achievement at grades, 4, 6, and 8 [73] and that assignment to a higher quality kindergarten classroom was associated with higher rates of college attendance and higher earnings in adulthood [74]. Although much of the discussion in early education focuses on prekindergarten, increased attention to kindergarten, and also the first three elementary grades, can broaden understanding of the goals of early education and the best ways in which to support children’s learning.

In conclusion, it is important to point out that results from this experimental evaluation are particularly notable in that the Tools of the Mind educational program is implemented without a high level of additional resources and support and using typical professional development activities. Teachers received typical levels of training and implemented the curriculum with materials that are well within the budget of the average kindergarten classroom. Given mixed findings in prior evaluations, however, further research is needed on the approach and its application across contexts. It will be particularly important in future analyses to identify the program’s “active ingredients.” This analysis is limited in that it focused only on the examination of the efficacy of the program without attention to fidelity of program implementation or specific program components. This analysis is also limited in that we conducted multiple statistical tests without correction and as a consequence there is the possibility that our results could have capitalized on chance. This concern is mitigated to some extent by the coherence of our findings and by the strong theoretical rationale for examining multiple interrelated aspects of children’s development. Further, although findings indicate that effects of Tools of the Mind are large on certain of the dependent variables in high-poverty schools, we had no hypotheses as to why some variables but not others would show this differential effect of the program. Despite these limitations, our findings do suggest that the adoption of readily implementable programs embodying a focus on self-regulation development in kindergarten can be expected to have substantial impact on reducing disparities in educational achievement while boosting early achievement for all children. As such, these findings have implications for educational policy at local and national levels. Specifically, they support the shift among school systems to investment in universal programs and assurance of high quality early education for all from prekindergarten through 3^rd^ grade [see Weiland and Yoshikawa [75] on efforts in Boston MA and the universal early education initiative in New York City as well as ongoing federal early education initiatives.] In contrast to earlier policy emphases on targeted, means-tested intervention supporting income-eligible children through programs such as Head Start, our findings suggest the value added from an integrated model of high-quality early education for all children across the early elementary grades.
